# Ways and means of coping with uncertainties of the relationship of the genetic blue print to protein structure and function in the cell

**DOI:** 10.1186/1478-811X-8-26

**Published:** 2010-09-17

**Authors:** Ernst JM Helmreich

**Affiliations:** 1 Weindl Lenzstrasse 19, 83727 Schliersee, Germany

## Abstract

As one of the disciplines of systems biology, proteomics is central to enabling the elucidation of protein function within the cell; furthermore, the question of how to deduce protein structure and function from the genetic readout has gained new significance. This problem is of particular relevance for proteins engaged in cell signalling. In dealing with this question, I shall critically comment on the reliability and predictability of transmission and translation of the genetic blue print into the phenotype, the protein. Based on this information, I will then evaluate the intentions and goals of today's proteomics and gene-networking and appraise their chances of success. Some of the themes commented on in this publication are explored in greater detail with particular emphasis on the historical roots of concepts and techniques in my forthcoming book, published in German: *Von Molekülen zu Zellen. 100 Jahre experimentelle Biologie. Betrachtungen eines Biochemikers.*

## Introduction

The formation of functional proteins in the cell depends on the reliable transcription and translation of the genetic information. Today, genetics aims at describing the functional correlate of the genome in the context of the living cell, in other words, it tries to build a bridge from genotype to phenotype. In classical genetics however, a gene was an abstract concept, a materially undefined entity that ferried a characteristic property from parent to offspring. Later, genes became chemically, structurally and functionally defined RNA or DNA polymers, [1], and chromosomes, where the genes are located in the cell, were shown to be tightly packed protein-ribonucleic acid complexes, containing a linear array of genes [2]. Finally, the syntax, the genetic message, was deciphered and shown to consist of words consisting of three letter syllables. Today, the question, what is a gene and how does it function [3] must be raised anew, a question that in the not too distant past would have seemed silly, because every schoolchild believed to know what a gene is. The root cause for this confidence came from the organisation of the genome in prokaryotes, where the gene and the product of its transcription, the mRNA, and the product of its translation, the resulting protein's amino acid sequence, were seen to be co-linear. This assumption was experimentally verified [4]. It also led to euphoric expectations in human genetics. After the human genome had been deciphered, the hope was to replace a diseased gene with a functional one, bringing, at the very least, the expectation of curing monogenetically-caused diseases. Co-linearity in prokaryotes is possible, because transcription and translation are not separated by cell compartmentalisation, as is the case for eukaryotes. In prokaryotes, the nascent genetic readout, the mRNA, binds directly to ribosomes where it is translated into protein. However, due to the spatial separation of genes and ribosomes, direct coupling of gene transcription, translation and protein biosynthesis is no longer possible in Eukarya. Whereas gene expression is confined to the nucleus, translation is an exclusively cytoplasmic event. Although separation of both processes makes gene expression much more complicated than in bacteria, it enabled the evolution of split genes and pre-messenger RNA splicing in Eukarya, which in turn led to a massive diversification of genetic information [5,6]. This, however, is only one of the key aspects that have profoundly changed our views. Most recently, advances in genetics have led to the comprehensive knowledge of the genome's output: the full extent of transcription and the regulatory role of the products of the non-coding DNA, the micro RNAs [7,8].

After the gene was shown to be composed of DNA, [9,10], its structure solved by Watson and Crick, [11,12], and its replication verified by Meselson and Stahl, [13], the next question that had to be answered was, how is the information in DNA transcribed and translated into the language of proteins? The direction of the search was set by Francis Crick's brilliant idea of a *unidirectional path, DNA→ RNA→ Protein. *Crick had suggested that *'...the main function of the genetic material, DNA, in a cell is to control, but not necessarily to direct the synthesis of proteins...' *thus hinting at a role of molecules, other than DNA, in protein synthesis. Moreover, on the same occasion [14], he pronounced his central dogma: *'*... *once information has passed from the nucleic acids into protein, it can not get out again, only transfer from nucleic acid to nucleic acid or from nucleic acid to protein is possible.... *(Citation from: Horace Freeland Judson's book, The eight day of creation. Makers of the revolution in Biology" [15]). Crick's dogma was a modern version of August Weismann's [16] in which the *'Keimplasma'*, containing the genes, was described as being separate from the cell soma, which contained everything else. A unidirectional transfer of information from DNA to protein would also have forestalled any kind of environmental influence on the gene, as Lamarck had envisioned, because proteins are the agents that communicate with the environment. While Crick's idea laid the foundations for the identification and characterization of the instruments of gene transcription and translation, tRNAs and mRNAs, and while he was right on transfer only from nucleic acid to nucleic acid, the idea of an unidirectional transfer of information, like any other dogma in biochemistry, was short lived. After Howard Temin [17] and David Baltimore [18] independently discovered reverse transcription from RNA to DNA, Crick's paradigm had to be abandoned.

A focal point of my discussion of the route from gene to protein is the plasticity of structure and function of some proteins, notably of antibodies and receptors [19] which allows one and the same protein to assume different structures and functions in the same cellular environment. I shall give examples of pliable and adaptable proteins and shall cite possible causes of ligand-induced malleability of proteins. Finally, I shall comment briefly on the revolutionary progress made in recent years in the design and improvement of new and old techniques respectively *in vivo *observations, advances that give me confidence that the ambitious goals of the *in vivo *21^st ^century biochemistry might be, at least in part, realized.

## Comments

### The genetics of today

The existence of split genes [20] was demonstrated by Richard J. Roberts und Philip A. Sharp [21]: Within exons, genomic regions containing coding sequences which are transcribed and translated into defined protein products, are embedded what were initially thought to be non-coding regions or introns. The splicing process leads to removal of the introns, and the remaining exons are fused and transcribed. Such a spliced gene may have contained many introns ranging from 40 to 100,000 nucleotides in length. Transcription of spliced genes results in a split RNA transcript. In the 1980s Thomas Cech and his colleagues observed that the processing and splicing of a 400 nucleotide long intron of the ribosomal RNA of *Tetrahymena thermophila *did not require any added proteins [22], leading to the conclusion that RNA itself can carry out splicing of its own RNA. The RNA of *Tetrahymena *is a ribozyme [23].

Splicing of ribosomal RNA, or rRNA, and splicing of pre-mRNA, are two different processes [1]. Removal of introns of pre-mRNAs and ligation of the remaining exons is executed by a nuclear machinery, the spliceosome. The spliceosome assembles itself anew on each intron of a mRNA that requires splicing. Consisting of four different, small nuclear ribonucleoproteins (snRNPs), and 100-150 additional proteins, the spliceosome constitutes one of the most complex cellular macromolecular machines identified to date.

Most mRNAs encoding more than one different protein can be spliced by the spliceosome in a manner, also known as alternative splicing, that ensures that different proteins can be generated from a single pre-mRNA. In this way, splicing contributes to the efficient and exhaustive use of the genetic repertoire and has thus been instrumental in facilitating the evolutionary process within organisms.

Alternative splicing also enables transcription to start at a gene and then continue transcribing another gene with another function or encoding a different protein, located remotely or even on another chromosome [24]. 4-5% of DNA, assigned to identified proteins is transcribed in this way.

Mobile or 'jumping' genes, were discovered in the 1950s by Barbara McClintock in corn, have also gained new relevance, one of the reasons being that they can come under different promoter control resulting in differential expression; a process shown to be responsible for cancerous cell growth [25]. One of the reasons why genes must be mobile is the compartmentalisation of gene transcription and the discrete location of genomic transcription factories, although the exact location of the latter remains uncertain. This makes movement of genes mandatory, because the number of transcription factories in each cell nucleus is limited. Consequently, genes located tens of megabases apart or even on different chromosomes must move to a transcription factory, in order to be transcribed [26].

Compartmentalisation of gene transcription, transcription-mediated gene fusion and genome-wide transcription, [27,28], and the implications of disorder in proteins [29] for regulation and protein interactions, will make it difficult for today's gene networkers to assign a coding region to a gene in a genome map. However, without such an assignment, it is problematic to link a protein to the gene that encodes it.

A further layer of complexity is contributed by mechanisms, some old, some new, that modify genes and chromosomes epigenetically [30], without permanently altering the inheritable sequence of the DNA. Moreover, not only is transcription more complex in eukaryotes and metazoa than in prokaryotes, the same holds for the translation of the message and its regulation. In recent years, we have learned that non-coding DNA [7] encodes small RNAs or micro RNAs [8] which control gene expression at the level of transcription and translation in metazoa and humans.

This short summary of the state of current genetics clearly illustrates that co-linearity of genotype and phenotype, of gene and protein, represents a simplistic interpretation of the situation in eukarya, and is only one side of the coin. The other problem for systems biology and proteomics, is the inherent intricacy of the path from the genetically-determined primary structure of a protein to its functional structure in the cell.

### From Gene to Protein

The optimistic expectation, that the genetically determined primary structure of a protein contains all the information required for formation of a unique, three-dimensional, secondary and tertiary, spatial, three dimensional structure was widely shared by my generation of biochemists. This is also what Jacques Monod had expected [31]. He thought that a protein has only '...*one defined structure and one unique communiqué...' *and indeed there are examples of where this expectation holds true.

#### I. Protein folding, the other half of the genetic code

Pauling's, [32] perception of the ways and means by which higher order structures of a protein are formed from the backbone structure of primary amino acids, guided Christian B. Anfinsen, [33] in his attempts to decipher the other half of the genetic code. He could show that de-natured ribonuclease, with its four -S-S- bonds reduced, spontaneously refolded into its native structure, once the denaturing agent, 8 M urea, was removed by dialysis and the scrambled enzyme exposed to a small amount of an -S-S- reducing agent, mercaptoethanol. Under these conditions, disulfide interchange eventually formed out of a mixture of 105 incorrectly folded isoforms, a single homogenous product which was indistinguishable from native ribonuclease. (Renaturation is driven by the free energy, ∆G, which is gained when the unfolded polypeptide chain refolds into a stable, native structure. The gain in enthalpy offsets the loss of entropy, when the more ordered, folded structure is formed).

Further supporting evidence came from the successful recombination experiments of Frederic M. Richards, [34], with ribonuclease S, a 20 amino acid fragment, cut off from the N-terminal half of ribonuclease, and the remaining core of the enzyme with its 104 residues, containing all four disulfide bridges of the native, parent molecule. The small and large fragment recombined correctly and the structure and function of the native ribonuclease were completely restored. The experiments of Frederic M. Richards proved that cooperative, non-covalent forces are also responsible for protein-protein recognition and interaction, just as Linus Pauling had foreseen [32].

Another way to solve the problem of protein folding rests on the expectation that it should be theoretically possible to predict the three-dimensional structure of a protein from the chemical and physicochemical properties of the constituent amino acid side chains and their behaviour in aqueous solution. However, although estimates of the forces involved in folding a protein have been made and continuously refined [35], all these efforts have been insufficient to permit reliable prediction of the higher order structures of larger proteins from their amino acid sequences.

Ribonuclease therefore turned out to be more of an exception rather than the rule. In many cases, one and the same protein can assume different conformations with different functions in the same cellular environment and location.

In the decades since Anfinsen's experiments, the instruments that help a cell to fold its proteins have been identified. Anfinsen and his colleagues [33] were already aware that folding may require assistance, because renaturation of ribonuclease *in vitro *took hours, much longer than the time needed for folding of a polypeptide chain of this length *in vivo*, where it is known to occur in about a minute or less. However, when Anfinsen and his colleagues [36] added an extract containing an enzyme which catalyzes disulfide interchange, renaturation occurred *in vitro *as fast as it did *in vivo*. In the meantime, a number of folding partners were discovered, including enzymes which help to form disulfide bonds and enzymes such as peptidyl prolyl isomerases which accelerate the cis-trans isomerizations of peptide bonds featuring a preceding proline residue [37,38]. The correct placement of disulfide bonds is often rate-limiting in protein folding, notably of proteins which are secreted. Some secreted proteins, for example trypsin, chymotrypsin [39] and insulin [40] are formed as inactive precursors, which are converted to the active form, with the right arrangement of disulfide bonds, by limited proteolysis. Moreover, cells that secrete proteins harbour disulfide isomerases that help to promote disulfide interchange and rearrangement of proteins to be secreted. Proteins which are secreted, or those targeted to the cell surface first enter the luminal space of the endoplasmic reticulum (ER) where they are properly folded and in some cases modified, for example, by glycosylation. Only properly folded and processed proteins can exit from the ER. Peter Walter and his colleagues, in San Francisco have studied the regulatory mechanisms in eukaryotic cells that control folding and secretion of proteins in the ER [41]. They concluded that in the event of misfolding, the unfolded protein response of the cell (UPR) comes into play, which can order the death of a cell with misfolded proteins by apoptosis. Folding malfunctions seem to play a role not only in familial protein folding diseases, but also in diabetes, cancer, viral infections and prion-based diseases [42].

The discovery and characterization of the heat shock proteins, such as HSP 60 and HSP 70, helped to gain new insights into the fate of proteins in the living cell. These and other chaperones accompany proteins in the cell from their formation to their degradation and removal. While without their help, newly folded proteins would form aggregates, this is only one of their many essential functions. By 1989, Walter Neupert's laboratory in Munich had described a role for a heat shock protein in the assembly of proteins in mitochondria [43]. Subsequently, chaperones were shown to help folding polypeptide chains in the course of their synthesis, to protect already folded proteins and to mark those which are incorrectly folded for degradation [44,45]. Chaperones may even participate in the folding process itself, like a folding enzyme [46].

Remarkably, chaperones are not only required for protein folding, they also help in the assembly of macromolecular complexes such as proteasomes, ribosomes and spliceosomes. For example, an assembly chaperone has recently been described that assembles the protein building blocks of the spliceosome and helps to build this complex machine in the cell [47,48]. This kind of chaperones probably assists in the biogenesis of all macromolecular complexes in the cell. (For more information see the review of Hartl, F. U. und Hayer-Hartl, M. in Science. (2002) [44], and the publication dealing with *cellular functions of molecular chaperones*, in: Mechanisms of Protein folding [49]).

Despite the significant body of knowledge that has accumulated relating to the mechanism of protein folding in the living cell, reliable folding of larger complex proteins in the laboratory remains to be achieved. As we learn more about ligand-induced conformational changes of proteins [50], it is becoming clear that the cause of our failure might be the ease with which some proteins are capable of changing their structure and function. Thus, the genetically-determined primary, back bone structure of a protein can in some cases form different higher order structures. This process is not only induced by binding of ligands, which regulate and determine the functionally-relevant actions and interactions of such proteins in the cell, because there are also intrinsically disordered proteins or parts of proteins.

### Flexible Proteins

Although Linus Pauling [51] had to abandon his instructive theory of antibody formation, his notion of a flexible secondary and tertiary structure of antibody binding sites in particular, and of proteins in general, has been vindicated.

#### I. The antibody: an example of a flexible protein

**Figure 1:**
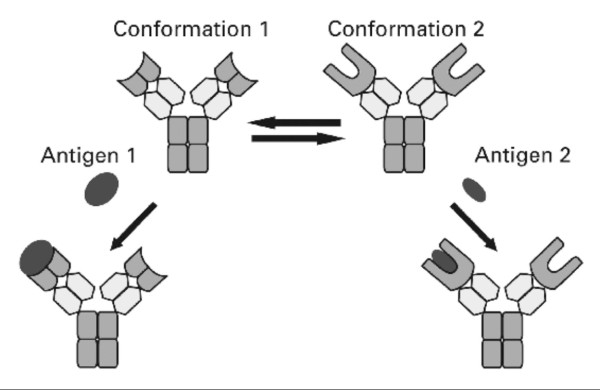
**Binding promiscuity of a monoclonal antibody**. One and the same monoclonal antibody has different binding sites for antigens, each of which can bind to structurally different antigens. The same antibody has two conformations, 1 and 2. Conformation 1 has binding sites with a shallow groove, which can accommodate even a protein antigen, (antigen 1). The same antibody can assume conformation 2 with a deeper binding site, which fits aromatic haptens, such as a DNP, a dinitrophenyl - hapten, antigen 2. Antibodies with such flexible, promiscuous, binding sites are in allosteric equilibrium. This scheme is a simplified version of a scheme of Foote, J. 2003. Isomeric Antibodies. Science. 229: 1327-1328) and was first shown with approval of the author and the AAAS in my forthcoming book in German: *<Von Molekülen zu Zellen. 100 Jahre experimentelle Biologie. Betrachtungen eines Biochemikers>*. It is shown here with permission of the GNT Verlag für Geschichte der Naturwissenschaften und der Technik. Diepholz. Stuttgart, Berlin.

Today, there is convincing evidence that antibody binding sites can accommodate structurally different haptens or antigens [52-54]. For example, a monoclonal antibody raised against the HIV-1p24 protein can bind to several unrelated peptides [55]. James, Roversi and Tawfik [56], from the Weizmann Institute in Rehovot have demonstrated that a monoclonal antibody, specific for a 2,4 dinitrophenyl (DNP) hapten also bound, with a broad range of affinities, to several, unrelated aromatic compounds and even to a protein. Adaptability of the antibody binding sites is also in agreement with different crystal structures of the same antibody, unbound and bound to a hapten. A fragment, Fv, of the variable binding site of this monoclonal antibody did crystallize, in the absence of the ligand, in two different, stable conformations and in complexes with aromatic haptens and with a proteinaceous antigen, it adopted a third and a fourth conformation [57]. Fig:1.

An antibody clearly illustrates the flexibility of a protein and the unpredictability of the structure-function relationship [58]. Thus, a monoclonal antibody exists in solution as an ensemble of isomers, each capable of binding a structurally-different ligand. The plasticity of antibodies is exploited in current medicine, notably of camelid antibodies (antibodies of camels), which are homologous to human VH, variable heavy antibody chains, and possess a simpler structure than other antibodies. Whether structural changes are limited to a spatial rearrangement of contact residues for the ligand in the antibody binding site, or are propagated allosterically to other parts of the antibody molecule remains to be determined. Israel Pecht [59], considering the high activation enthalpies for binding hapten or antigen, is inclined to assume that hapten-binding leads to a large, long-range, conformational transition of the whole antibody.

#### II. Receptors are examples of flexible proteins, demonstrating the advantages of structural plasticity and functional versatility for regulatory proteins

In the first sentence of the preface of his unsurpassed treatment of allosterism, Max Perutz praised the versatility of proteins [60]: *'In the popular view the structure of DNA has told us all about the molecular basis of life, but in fact DNAs and most RNAs are chemically inert, whereas proteins are the workhorses of the living cell. They function as catalysts and genetic regulators, pumps and motors, receptors and transducers, stores and transporters, scaffolds and walls, toxins and antitoxins, conductors and insulators, and much more'.*

Aside from antibodies, receptors are prime examples of ligand-induced adaptability of structure and functional versatility. However, receptors also exemplify the functional advantages of structural plasticity and functional versatility. Some receptors can bind different ligands and perform different actions. Promiscuity of ligand coupling is biologically important, because it enables, for example, one and the same cytokine to cross-talk to different receptors, thus allowing one and the same cytokine to control different biological pathways. This considerably enlarges the signalling repertoire of a given cytokine.

Receptors are proteins engaged mostly in signal transfer from the outside to the inside of and between cells [61]. Aside from receptors in the plasma membrane of cells, there are receptors bound to intracellular boundaries, to mitochondrial and nuclear membranes. Nuclear receptors transmit signals from steroid and non-steroidal hormones such as thyroid hormones and retinoic acids, which can pass through the plasma membrane. Finally, pathogenic microbes [62] may transfer their receptors into cells, making them susceptible to infection [63].

Like receptors, receptor ligands have many diverse structures and functions acting as sensory signals, odorants and photons, hormones, growth factors, cytokines and chemoattractants, and much more. Ligands may be as small as a photon or as large as a protein, such as insulin. In some cases, the interaction with ligands causes a structural transition of the receptor, which triggers tyrosine phosphorylation. Some receptors have intrinsic tyrosine kinase activity and phosphorylation sites on the cytoplasmic side of the receptor chains, others recruit tyrosine kinases when activated and, in other cases, activation of a receptor and signalling requires the recruitment of a co-receptor.

**Figure 2:**
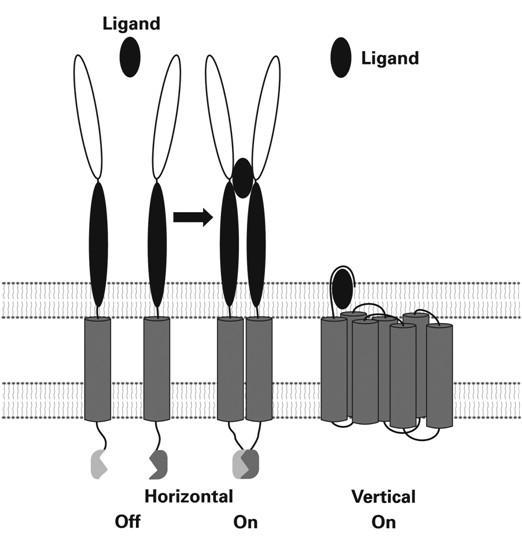
**Key characteristics of horizontal and vertical receptors**. The differences in structural changes on activation by binding the ligand are indicated schematically. The striking feature of horizontal receptors is that they associate and form dimers or multimers upon activation, whereas activation of vertical receptors does not involve quaternary structure changes of the already associated, preformed oligomer. This figure was originally reproduced from R.M. Stroud and J. A. Wells. 2004. Mechanistic Diversity of Cytokine Receptor Signalling across Cell Membranes, and the journal, Science STKE.2004.Re: 7, pps: 1-17, with approval of the authors and the AAAS in my forthcoming book in German: *<Von Molekülen zu Zellen. 100 Jahre experimentelle Biologie. Betrachtungen eines Biochemikers>*. It is shown here with permission of the GNT Verlag für Geschichte der Naturwissenschaften und der Technik. Diepholz. Stuttgart, Berlin.

**Figure 3:**
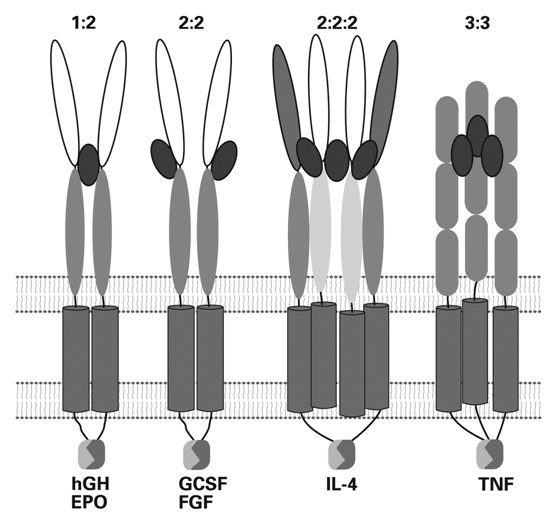
**Illustration of the variety of different multimeric assemblies of horizontal cytokine-receptors on ligand binding and activation**. hGH = human growth hormone. EP0 = erythropoetin. GCSF = Granulocyte Colony-stimulating factor. FGF = Fibroblast growth factor. IL-4 = Interleukin 4. TNF = Tumour necrosis factor. This figure is originally reproduced with permission of the authors R. M. Stroud and J. A. Wells (2004) and the AAAS from *<Mechanistic Diversity of Cytokine Receptor Signalling Across Cell Membranes>*, and the journal, Science STKE.2004.Re: 7, pps: 1-17, and is shown in my forthcoming book in German: *<Von Molekülen zu Zellen. 100 Jahre experimentelle Biologie. Betrachtungen eines Biochemikers>*. It is reproduced here with permission of the GNT Verlag für Geschichte der Naturwissenschaften und der Technik. Diepholz. Stuttgart, Berlin.

Robert M. Stroud and James A. Wells [64] draw the distinction between two kinds of receptors. In one kind, ligand-induced structural changes bring about lateral association of separated subunits in the membrane, leading to dimerization or oligomerization of the receptor. In the other kind, structural changes are confined to a re-arrangement of a pre-existing polymeric assembly. Stroud and Wells have named receptors belonging to the first group, horizontal receptors and those belonging to the latter group, vertical receptors. Though I have adopted their classification here, there are some receptors that do not fit into their scheme. Fig: 2 and Fig:3.

Horizontal receptors bind mono-or multimeric protein ligands. This kind of receptors control gene transcription and translation and regulate cell replication, growth and cell death. The three-dimensional structures of almost a dozen complexes of the ligand-binding extracellular parts of these receptors with their ligands have been solved [65]. Although the structural rearrangements on binding of the ligand differ considerably from one receptor to the next, as Walter Sebald et al. have shown [66-68], all horizontal receptors eventually form dimers or oligomers on ligand binding and activation. A consequence of receptor oligomerization is the formation of new interactive surfaces on the inside of the membrane for coupling partners, mostly components of intracellular signalling chains. The best-characterized examples of horizontal receptors are members of the family of human growth hormone receptors (hGHRs). The structural flexibility and coupling versatility of both receptor and ligand is startling. The same structures in human growth hormone can bind to different receptors [69], and, vice versa, hGH receptors can use the same structural elements for binding structurally different ligands.

Consisting of a modular structure and accepting both intra-and extra-cellular signals, the integrins constitute another group of horizontal receptors with remarkable structural pliability and functional versatility. Upon phosphorylation by tyrosine kinases, the tails of integrins bind specifically to muscle proteins such as talin, and transmit signals to the submembranous cytoskeleton [70,71], whereas the extracellular domain of integrins binds to constituents of the extracellular matrix. In this way, integrins modulate cell-cell interactions [72] and play a central role in cell-cell adhesion.

The largest family of receptors is the species of seven transmembrane, heptahelical, receptors, coupled to G proteins, (G = GTP-binding). One cannot assign these receptors unequivocally to the vertical receptor class, because there is increasing evidence of oligomerization of G-protein-coupled receptors [73]. These receptors are ubiquitous: they are found in the cell membranes of archeae, halobacteria and metazoa, as well as plants, mammals and humans. Rhodopsin, located in the retina of the eye, which is activated by light [74], bacterio-rhodopsin [75], opsin [76] and the ß_2_-adrenergic receptor [77] also belong to this group. These are currently the best characterized receptors at the structural level and are also targets of modifiers and inhibitors with important therapeutic value in medicine, particularly cardiology. These receptors control ion pumps, phosphorylation-cascades and cell metabolism. One way, but not the only way, to transmit signals from these receptors after activation is through G proteins. For more information, see [19,78-80] and the recent excellent review by Ilka Böhme and Annette G. Beck-Sickinger in this journal [81].

The flexibility and functional versatility of antibodies and receptors raise the question of how a protein must be designed in order to be able to drastically change its structure and function on binding ligands. New insights into the dynamics and physicochemical properties of some proteins may help to answer this question. The dynamics of proteins may also explain why protein-folding *in vitro *has turned out to be such an intractable problem.

### Internal Dynamics of Proteins

**Figure 4:**
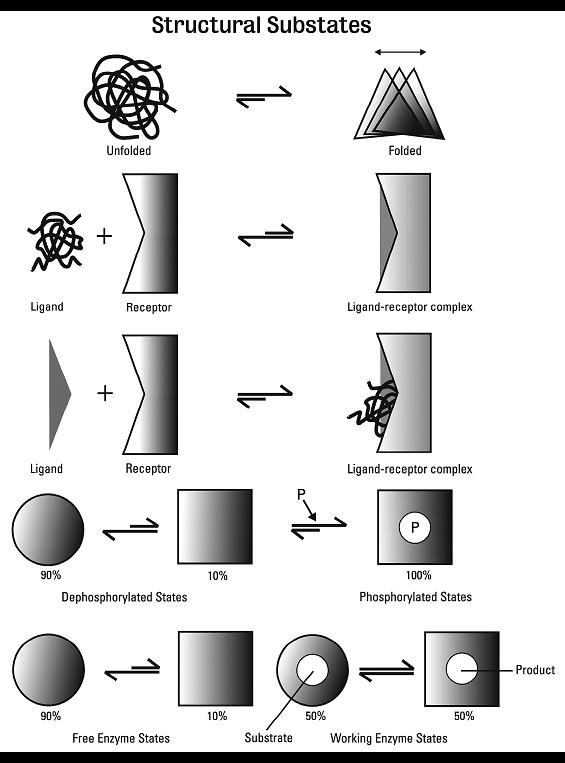
**Examples of processes involving protein dynamics**. From the top row: (1) Conformational changes between folded and unfolded states, (2) ligand receptor interactions, (3) a representation of the effects of covalent modification of a protein, (4) the role of phosphorylation, (P), in stabilising one of several interconvertible forms and finally (5) transitions between structural sub-states of enzymes, on going from an inactive to an active, substrate-bound state are shown. The double arrows connect the interconvertible states with the longer arrows indicating the energetically favoured direction of the change. Percentages, where listed, give rough estimates of how much each state is populated. This is a simplified version of the scheme of Yuanpeng J. Huang and Gaetano T. Montelione (2005). News and views: Structural Biology: Proteins flex to function. Nature, 438, 36-37. It was reproduced with approval of the authors and Macmillan Publishers Ltd. in my forthcoming book in German: *<Von Molekülen zu Zellen. 100 Jahre experimentelle Biologie. Betrachtungen eines Biochemikers>*, and is shown here with permission of the GNT Verlag für Geschichte der Naturwissenschaften und der Technik. Diepholz. Stuttgart, Berlin.

Figure 4 lists examples of processes in which protein dynamics play a role. Hans Frauenfelder [82], a pioneer in the field of protein physico-chemistry, described the dynamics of a protein, myoglobin, as early as the 1970s. According to Hans Frauenfelder et al., [83], some proteins are an assemblage of conformational variants which move in an intricate, multidimensional energy landscape and interconvert from one structure to another in tens of femtoseconds (A femtosecond is 10^-15 ^sec) or hours. A detailed analysis of the temporal order of the conformational changes may eventually shed more light on the topography of the energy landscape and explain the properties of these proteins [84]. Since individual molecules may follow different paths, single-molecule methods are now increasingly used. Analysis of this intricate problem is being actively pursued and progress in numerical simulations is already permitting increasing complexity to be handled [85,86].

NMR spectroscopy, has also provided structural information on proteins exhibiting internal dynamics [87-89]. (For a review see: [[90]). For example, Eisenmesser et al. [91,92] studied the structural changes in the enzyme cyclophilin A, on going from a non-active to a catalytically active state. (Cyclophilins are prolyl isomerases, that are inhibited by cyclosporin [93]). Eisenmesser et al. observed that during catalysis some amino acids can change their position as fast as the elementary steps of the catalytic reaction, suggesting that conformational changes of the enzyme are synchronized with catalysis. However, surprisingly, identical structural changes also occur in the non-active enzyme. The only difference between the active and inactive enzyme is that the sub-states are differentially populated. One might speculate that proteins with an intrinsic plasticity such as cyclophilin A may have been earmarked by evolution to become enzymatic catalysts, because all the structural prerequisites for catalysis are already present in the non-active protein.

Another protein, for which a comprehensive range of NMR data are available is human ubiquitin, a relatively small protein of 76 amino acids. The native state of ubiquitin was described [94] as an ensemble of different conformers, interconverting on a picosecond to nanosecond scale. The mobility of the structure of this protein is not restricted to its surface loops and side chains, it also includes its core, which was described as liquid-like and fluctuating [95,96]. It is remarkable that changes including mutational changes, leading to a complete redesign of its hydrophobic core do not compromise the structural integrity of this protein. According to Martin Karplus and his colleagues [97], such properties may even be representative of proteins in general.

The bewildering variety of conformational sub-states of some proteins is an indication of their structural plasticity and versatility of function. We are just beginning to understand what makes some proteins flexible and what kind of forces drive structural transitions. The extraordinary malleability of this kind of proteins is also of great interest for protein engineers trying to redesign proteins in the laboratory.

The progress in structural analysis and, particularly, NMR spectroscopy, has significantly extended our insight into co-and post-translational modifications in the last decade, a theme which has already been reviewed exhaustively in recent years [29,98,99]. In this context, it suffices to say that the diversity and versatility of structural disorder of proteins and of protein modifications, in addition to the consequences of rRNA and mRNA splicing, has greatly increased the repertoire of possible protein structures and functions, way beyond the information deducible from the genome. Disorder in proteins has important consequences, notably for the function of regulatory proteins, but also for protein interactions in the cell. It remains to be seen to what extent systems biology and proteomics will be able to handle the structural and functional versatility of proteins, notably of proteins carrying out regulatory functions.

In conclusion, structural flexibility seems to be an inherent property of some, if not all, proteins. It allows versatility of function and is essential for regulation. Such properties can not be deduced from the genetic readout, from the primary back bone structure of these proteins. Moreover, in many cases, regulated proteins can change their structure and function in the cell as a consequence of reversible covalent modifications, such as by phosphorylation and/or other changes. About 2% of human genes encode protein kinases and phosphatases. Such reversible modifications also leave no traces in genetic readouts.

Another aspect that cellular biochemistry must deal with is the role of the environment. Compared with hemoglobin and other water soluble, allosterically, ligand-regulated proteins, the information on structural transformations of membranous receptors on ligand-binding and activation is limited. The reason is that receptors are embedded in a water-lipid interface. This makes it difficult to isolate them in a native conformation and identify the structures of the active conformers at high resolution. Therefore, the structural information at hand often encompasses only the water soluble extracellular part of the receptor, and does not allow assignment of specific roles in signal transmission to reactive interfaces on the cytosolic side, which are exposed to the cell's interior, where coupling with signal transmitters occurs. (Notable exceptions are the heptahelical G-protein coupled receptors).

### The role of the environment

Although I shall concentrate here on the role of the membranous environment for receptor structure and function, the influence of the environment on actions and interactions of proteins, deserves, of course, serious consideration in all cases, because environmental influences on structurally variable proteins are not restricted to the membrane and are most likely also responsible for configurational rearrangements of proteins in other cellular environments.

How can the membranous environment affect receptor structure and function? [100]. The membranous environment may affect receptor activation and de-activation, signal transduction and receptor re-cycling in many ways [101]. Membrane lipids could either influence receptors through global changes in the physical state of the water lipid bilayer, for example through microviscosity changes, or individually through discrete and specific interactions. While, the former is unlikely in homoiothermic animals, the latter is to be expected. Although, the central importance of this problem is widely recognized, we still do not have enough data on the physicochemical restraints that individual membrane lipids may exert on the mobility and reactivity of proteins embedded in the membrane. An example is the role that is being attributed to complexes, or transducisomes, containing whole signal transmission chains, arranged on lipid scaffolds in the membrane [102,103]. Although, this is undoubtedly an attractive perspective, the role of lipids in forming and maintaining such transducisomes in membranes is not yet clear. We neither know the composition, size or the lifetime of such lipid-protein complexes [104].

Although, it is evident that the behaviour of proteins at the water-lipid interface is difficult to study, I also recall that for the older generation of biochemists, the lipid bilayer was mostly a undefined mixture that is better circumvented. For example, Carl F. Cori, [105], one of the great biochemists of the 20^th ^century, with whom I had the privilege to work with in the 1950/60s, although finally accepting a role of insulin in making the membrane permeable for sugars [106], insisted that an intracellular enzyme, glucokinase or hexokinase, rather than a membrane-bound insulin receptor activating a membranous glucose carrier, controls the uptake of glucose into muscle.

The increasing awareness of the important role of the membranous environment in reactions of pivotal importance for the life of cells, brings hope that in the future more biochemists will try to decipher the influence of this particular solvent milieu on the many important reactions that occur in or at water-lipid interfaces in the cell. I expect that an important incentive for such greatly needed studies will be the availability of new methods for *in vivo *observations, methods that allow visualization of proteins in their natural environment and study of their movements and interactions *in situ.*

### New and old ways of *in vivo *observation

Already in the 1960s, biochemists of my generation, notably those who were interested in regulation, were keen to determine the cellular location of the enzyme or the protein they were studying. However, cellular compartmentalisation made any extrapolation of the *in vitro *to the *in vivo *situation speculative and tentative. I described the situation in 1969 [107]^.^: *'Equally as important as the actual molecular mechanism of allosteric transitions which may differ from enzyme to enzyme, is the in vivo expression of the regulatory potentiality of enzymes which control multienzyme sequences. The regulatory behaviour of an enzyme in the isolated state can only serve as a guide which may hopefully lead us to its actual control properties in the living cell'. *At that time, one tried to localize metabolites in cells by using radioactively labelled 2-deoxyglucose which, after being phosphorylated to 2-deoxyglucose-6-phosphate, accumulates in the cell [108]. Because it can not be metabolized further, this compound could be localized by a combination of radioautography and electron microscopy and from its position in the cell, one could deduce the location of the kinase which had phosphorylated the sugar analogue [109]. Later, receptors were labelled with fluorescent dyes [107] and their movements in cells studied [110]. Today, the introduction of a gene for fluorescent aquaporin into a living cell and its fusion to a gene expressing the protein to be localized, has led to new and improved methodologies to monitor a protein and visualize its position in the intact cell [111,112]. Roger Tsien's methods in conjunction with other physical methods, such as fluorescence energy transfer, FRET, fluorescence recovery after photobleaching, FRAP, and fluorescence correlation spectroscopy with single molecule detection, [113], today allow the identification and tracking of an increasingly wide range of molecules, both large and small, in their natural environment. The introduction of new and refinement of older methods has already brought us a wealth of new and valuable information on receptor function in living cells [114-116]. In the future, the aim is to enlarge the number of proteins that are visualized until one gets a *movie*, showing movements and interactions of all kinds of proteins in a living cell [117].

Spectacular advances have also been made in optical microscopy by Stefan W. Hell in Göttingen [118,119]. Until now, the Abbe diffraction barrier of optical microscopy limited the resolution of conventional light microscopy to 200 to 300 nm in lateral dimensions, leaving many intracellular organelles and molecular structures unresolvable, thus limiting the use of microscopy for three-dimensional imaging of biological structures. Now however, the *Abbe *diffraction limit has been transcended, and resolutions of 20-30 nm and 50-60 nm in the lateral and axial dimensions respectively have been achieved. With new techniques [120], such as stochastic three-dimensional optical reconstruction microscopy, STORM, a resolution can be obtained which enables the visualization of cellular structures by light microscopy in three dimensions.

Equally impressive are the advances in recent years in the development of cryoelectron microscopy and cryoelectron tomography. Following the pioneering work in the 1960s of De Rosier and Aaron Klug, cryoelectron tomography is now beginning to allow to make visible complex molecular assemblies within a living cell, such as actin bundles, intermediate filaments, which build the scaffold of a cell. Moreover, one can monitor proteins and nucleic acids on ribosomes, the cellular sites of protein synthesis [121,122]. In the future, all will depend on how far one will be able to increase the resolution, which is still in the tenth nm range.

Attempts are also being made today to study the structure of proteins in the living cell by in cell NMR spectroscopy [123,124]. This might be the beginning of a new era of structural analysis of proteins *in situ. *Another technical development that deserves attention, is the application of high resolution scanning X ray diffraction microscopy, or SXDM, to *in situ *objects [125,126].

## Conclusions

### The chances of today's functional genetics and proteomics

Today, it is agreed that a functional assignment of the genome is needed [127]. For that purpose, the ENCODE project, ENCyclopaedia Of DNA Elements) [128], which should provide functional assignments to all genes in the human genome has been organized and implemented. The most advanced sequencing techniques, combined with the most sophisticated computational methods are being applied. A pilot study, covering 1%, or 30 megabases, of the human genome, showed that nearly all of the DNA in a human chromosome is transcribed into RNA, as well as revealing the existence of many, still unrecognized, stretches of DNA with as yet unknown functions.

Another objective lies in finding out how the expression of DNA, packed in the chromatin of the chromosome, is regulated and epigenetically controlled. This is a sign that in the future, in addition to all the efforts aiming at a functional definition of the genome, one will also take into account the bewildering and still growing number of ways and means of how the genetic readout is modified, controlled and regulated. Depending on how cell-specific gene expression is, one might even have to identify the functional role of genome sequences in individual, differentiated cells.

Stefan Bornholdt, [129], has pointed out that going from measurements of all the factors, including all regulatory factors, that are responsible for the expression of a single gene, to measurements of the same parameters controlling the expression of hundreds and thousands of genes in a gene network, would make the number of differential equations that have to be solved to describe such a realistic situation intractably large, at least for the time being. But, mathematical integration is necessary to handle large amounts of data and model complex biological systems. Such concerns are realistic, and have also been raised by the physicist and evolutionary biologist, Alfred Gierer [130]. Thus, it seems that today's systems biology must, like Odysseus, guide its ship with care and imagination, avoiding the perils of Scylla and Charybdis. On the one hand, large amounts of data are necessary to describe complex biological processes in living systems and on the other, it may turn out that the use of this plethora of data is limited by the availability of algorithms capable of integrating them. This will be a difficult problem to solve for mathematicians and computer specialists.

**Figure 5:**
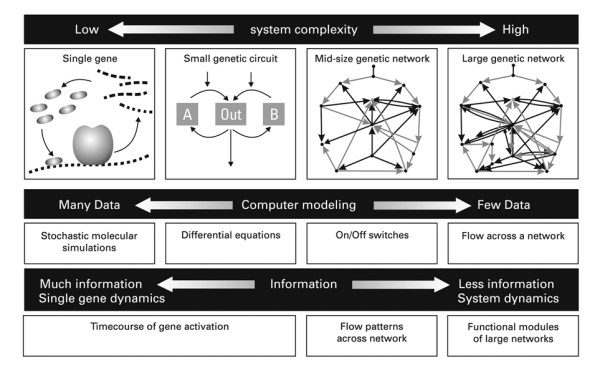
**The relationship between system complexity and the present modeling possibilities**. Whereas single genes can be modeled in molecular detail by stochastic simulations, (Top: left side), differential equations are already required for modeling small genetic circuits (centre left). Midsize genetic networks can at present only be modeled, when simplified, by concentrating on on-off switches, and large genetic networks are for the time being still out of reach of predictive simulations and modeling. Finally the last row shows that much information is available in the case of the consequences of the activity of single genes. The available information then gradually decreases from flow patterns of the activity of gene networks to functional modules of large networks. Activity patterns of large genetic networks are for the time being, still out of reach of predictive simulations and modeling. This is a simplified version of an illustration which indicates the different complexity of systems by Stefan Bornholdt, (Stefan Bornholdt, (2005). Less Is More in Modeling Large Genetic Networks. Perspectives: Systems biology. Science 310, 449-451). It is shown with permission of the author and the approval of Macmillan Publishers Ltd., in my forthcoming book in German: *<Von Molekülen zu Zellen. 100 Jahre experimentelle Biologie. Betrachtungen eines Biochemikers>*. It is reproduced here with permission of the GNT Verlag für Geschichte der Naturwissenschaften und der Technik. Diepholz. Stuttgart, Berlin.

The relationship between system complexity and the present modelling possibilities is shown in Fig: 5.

The obvious way to deal with such immense complexity, is simplification and reduction, although there are cogent reasons for which one has had to turn, for example, from single genes to gene networks, because deletions or mutations of single genes often have no discernible effect on the cell. For the time being however, I do not see any other way. However, here again a word of caution is warranted: for any kind of reduction, the question is of course how far can one reduce a complex biological process, without losing characteristic features? There are different ways to reduce complexity. One possibility is to choose a simple object. This is exemplified by Sidney Brenner's [131] introduction and successful use of *Caenorhabditis elegans *in genetics. The recent discovery of RNA-interference in *Caenorhabditis *by Craig Mello und Andrew Fire [132] demonstrates the value of a simple experimental object. Although, these experiments show the value of a suitable model organism for experiments in biology, one should not forget that the success of using simple biological objects, depends on the kind of questions asked.

Another possibility to simplify metabolic and genetic networks is to focus only on on/off switches and feedback loops that control whole systems [133]. Rather than studying complex sequences of reactions, one concentrates on the pacemaker of a chain of reactions. An example is the control of a chain of metabolic enzymes, for example glycolysis, by a single enzyme, serving as pacemaker [134], or the control of many genes in an operon by a promoter. These stripped down models may not represent the much more involved dynamics of the actual system in the living cell, but by comparing such simplified models with the actual situation in the cell, one can at least see whether and when, and to what extent, such binary on/off switch models mimic the dynamics of the real complex network in the living cell. For example, such simplified models, could correctly predict the dynamics of the complex genetic network, controlling segment polarity in the development of *Drosophila melanogaster *[135], and a simplified binary model of the genetic network controlling the yeast cell cycle [136] realistically modelled the actual situation.

I have commented on some of the problems functional genetics and *in situ *proteomics will face in the future. The extent to which the new *in vivo *experimental biology of the 21^st ^century will be able to handle these challenges is yet to be seen. The solution of these and other problems will depend on whether we shall be able to handle, both the stochasticity of gene expression and the complexity of the genetic readout. This requires large amounts of reliable *in vivo *data and algorithms, capable of transforming huge amounts of data into realistic models. Despite all uncertainties and all the problems that remain to be solved, there is no doubt that a new era has begun. On the one side, today's systems biology is part of and profits from today's exponentially growing information technology, that has already proved its ability to handle large amounts of data. (Google and similar search engines are examples). But, systems biology and *in vivo *proteomics also represent a new experimental biology, for which the living cell and its functions have become research objects. To study processes in the living cell, old techniques, such as microscopy have been refined and new techniques developed. The exponential and continuing progress in the development of techniques in the last years, and the optimism in handling complexity [137] make me optimistic that some, but not all, of the ambitious goals of systems biology and proteomics may be attained. The new area today reminds me of the beginnings of cell biology in the first half of the 19^th ^century, which the microscope in the hands of the pioneers of cell biology, of Rudolf Virchow, [138] and of O. and R. Hertwig, [139,140], helped to make possible.

*In vivo *observation of the molecular organisation of living systems will do away with the persuasive argument that the insights obtained by biochemists from *in vitro *studies are uncertain and tentative, because living systems can only be observed under conditions in which their integrity is preserved. This view was shared by Niels Bohr [141] (1885-1962. Nobel prize for Physics, 1922), who is probably the best-known example of a famous physicist interested in biology. (An interest that may have had its origin in the fact, that his father was a renowned physiologist). The functions and the material properties of a living organism were for Bohr only complementary and could not be derived from one other, a view still shared today by some chemists and physicists. Would Niels Bohr still be alive, he would certainly welcome the new *in vivo *biochemistry of the 21^st ^century.
